# Office Hysteroscopy for Infertility: A Series of 557 Consecutive Cases

**DOI:** 10.1155/2010/168096

**Published:** 2010-04-14

**Authors:** Martin Koskas, Jean-Luc Mergui, Chadi Yazbeck, Serge Uzan, Jacky Nizard

**Affiliations:** ^1^Department of Obstetrics and Gynecology, Hôpital Bichat, 46, rue Henri-Huchard, 75018 Paris, France; ^2^Department of Obstetrics and Gynecology, Hôpital Tenon, Paris, 4, rue de la Chine, 75020 Paris, France; ^3^Department of Obstetrics and Gynecology, Hôpital Pitié, 47-83 boulevard de l'Hôpital, 75013 Paris, France

## Abstract

*Objective*. To study incidence of abnormal hysteroscopic findings according to age. *Methods*. We retrospectively studied 557 consecutive office hysteroscopies in patients referred for incapacity to conceive lasting at least 1 year or prior to in vitro fertilization. Rates of abnormal findings were reviewed according to age. *Results*. In 219 cases, hysteroscopy showed an abnormality and more than a third of our population had abnormal findings that could be related to infertility. Rates of abnormal findings ranged from 30% at 30 years to more than 60% after 42 years. Risk of abnormal finding was multiplied by a factor of 1.5 every 5 years. *Conclusion*. Our data are an additional argument to propose office hysteroscopy as part of first-line exams in infertile woman, regardless of age.

## 1. Introduction

Hysteroscopy is the gold standard procedure for uterine cavity exploration [1]. However, the World Health Organization (WHO) recommends hysterosalpingography (HSG) alone for management of infertile women [2]. The explanation for this discrepancy is that HSG provides information on tubal patency or blockage. Office hysteroscopy is only recommended by the WHO when clinical or complementary exams (ultrasound, HSG) suggest intrauterine abnormality [3] or after in vitro fertilization (IVF) failure [4]. Nevertheless, many specialists feel that hysteroscopy is a more accurate tool because of the high false-positive and falsenegative rates of intra uterine abnormality with HSG [1, 5, 6]. This explains why many specialists use hysteroscopy as a first-line routine exam for infertility patients regardless of guidelines. The aim of this retrospective study is to describe hysteroscopy findings in a population of 557 infertile patients.

## 2. Materials and Methods

We analyzed retrospectively 557 patients referred for hysteroscopy for incapacity to conceive lasting at least 1 year or prior to IVF, from November 2002 to July 2006. This population represents one third of hysteroscopies on that period. All hysteroscopies were performed by the same operator (JLM).

Procedures lasted approximately two minutes without anesthesia or cervical preparation in an office gynecology setting. Diagnostic video-assisted hysteroscopy was performed using a flexible hysteroscope (flexible hysteroscope, Olympus HYF-P, Paris, France) with an outer diameter of 3.1 mm. Procedures were not video recorded. The uterine cavity was expanded under manual hydrostatic pressure (saline solution). Hysteroscopy was performed with a standard sequence, inspecting the endocervical canal, uterine cavity, endometrium, and tubal ostia. Findings were recorded using a standard report.

Statistical analyses were performed using Stata 9.2 for Windows (StataCorp LP, TX, USA) and Statistica 6.0 (StatSoft, OK, USA).

## 3. Results

On 557 successive patients, hysteroscopy could not be performed in one case because of pain. We observed no perforation, hemorrhagic, or metabolic complications. The mean age at hysteroscopy was 35.3 years (21–44 years).

Indication for hysteroscopy was pre IVF in 78.8% (439/557) and infertility in 21.2% (118/557). Women investigated with hysteroscopy were nulliparous in 73.4% (409/557), primiparous in 21.4% (119/557), and multiparous in 5.2% (29/557).

Hysteroscopy was normal in 60.5% (337/557) and among women with abnormal results, 20% showed more than one abnormality (44/220).

### 3.1. Cervico-Isthmic Abnormalities (Figure 1)

Cervico-isthmic abnormalities were present in 4.3% (24/557) of patients with 13 cases of polyps (2.3%), 9 stenosis (1.8%), 2 adhesions (0.4%). The stenosis was complete and did not allow to complete the procedure in 4 cases.

### 3.2. Uterine Cavity Abnormalities (Figure 2)

Uterine cavity was seen and normal in 72% of the cases (401/557). It could not be explored in 4 cases because of complete cervical stenosis.

Observed abnormalities were the following.

- *Adenomyosis aspect*: 17 cases (3.1%). Images compatible with adenomyosis were small openings in the endometrial surface, dark blue colour cystic lesions, rigid, tight tubal ostium (erecta), or T form uterus.
- *Intrauterin adhesion (IUA), synechiae: *22 cases (3.9% of all hysteroscopies).
- *Septate uterus: *4 uterine septa (0.7% of all hysteroscopies).
- *Hypoplasia and uterus unicornis: *15 hypoplasia (2.8% of all hysteroscopies), uterus unicornis in 2 cases.
- *Sub mucous myoma: *13 cases (2.3% of all hysteroscopies).
- *Deformed cavity from intramural myoma: *17 cases (3.1% of all hysteroscopies); unique (10 cases), multiple (7 cases).
- *Endometrial polyp: *54 cases (9.7% of all hysteroscopies). Unique in 30 cases (5.4% of all hysteroscopies). Their location was corporeal (19 cases) or cornual (11 cases). Multiples polyps were observed in 4.3% of all hysteroscopies (24 cases).
- *Trophoblastic retention: *7 cases (1.3% of all hysteroscopies) showed images compatible with a trophoblastic retention. Criteria used were a previous pregnancy with miscarriage, no ultrasound control of uterine vacuity, and a typical macroscopic aspect. Those were localized in the utero tubal junction (4 cases), or occupying the whole uterine cavity (one case).

**Figure 2:**
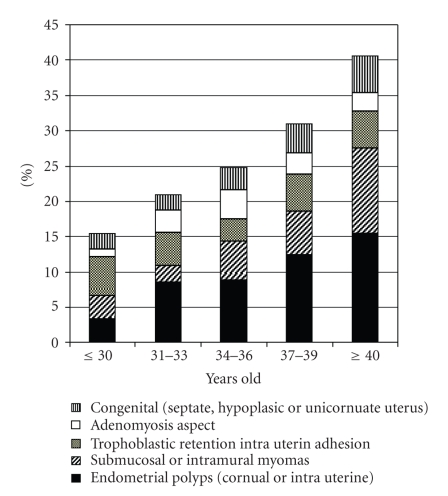
Rates of intrauterine abnormalities in 557 infertile women during office hysteroscopy according to age.

Findings according to age are given in Figure 2.

### 3.3. Ostial Abnormalities

A tubal ostium could not be seen in 15 cases, due to cornual adhesions (2 cases), trophoblastic retention (1 case), intra uterine adhesion (6 cases), inflammation (2 cases) or minor hemorrhage (1 case), endometrial hyperplasia (1 case), or unicornuate uterus (2 cases).

Both tubal ostia could not be seen in 9 cases, due to cornual adhesions (2 cases), retention (2 cases), intra uterine adhesion (1 case), inflammation (2 cases) or minor hemorrhage (1 case), and endometrial hyperplasia (1 case).

### 3.4. Endometrial Abnormalities

**Figure 3:**
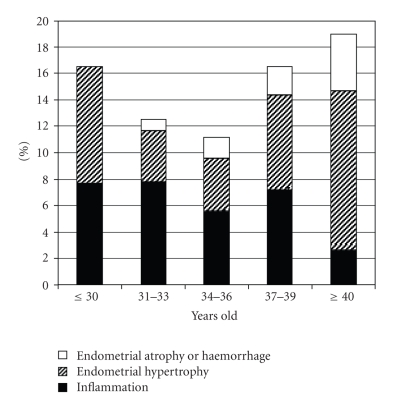
Rates of endometrial abnormalities in 557 infertile women during office hysteroscopy according to age.

Findings are given in Figure 3.

Endometrial inflammation was characterized by the presence of areas of red endometrium flushed with a white central point, localized or scattered throughout the cavity.

**Figure 4:**
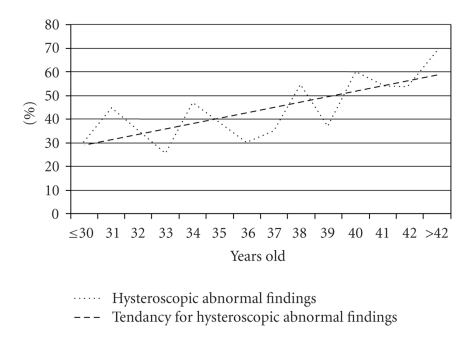
Rates of abnormal findings in 557 infertile women during office hysteroscopy according to age.

Continuous analysis with logistic regression shows that the risk to have an hysteroscopic abnormal finding increases with age (*P* < .001, OR = 1.076, IC = 1.04–1.12). Each year the risk is multiplied by 1.08. The risk is therefore multiplied by a factor of 1.5 every 5 years (Figure 4).

## 4. Discussion

We found that first-line office hysteroscopy for infertility shows abnormal findings in 40% of woman. This proportion increased with age, ranging from 30% at 30 years to more than 60% after 42 years. These findings are based on a large cohort of infertile women, with homogeneous age distribution. Hysteroscopies were performed consecutively by a single-trained operator. All investigations were performed using a flexible minihysteroscope which provides high patient acceptance since it makes hysteroscopy a painless and well-tolerated procedure. However, symptoms, clinical examination, ultrasound findings, HSG or hormonal blood sampling results characteristics were not available in our population. Moreover, there was no control group of fertile women to compare our findings with. Patients were referred from many hospitals and private clinics, with no homogeneity in infertility investigations prior to hysteroscopy. Finally, the absence of video recording did not allow control of findings by a different operator. No possibility of re-evaluation of the findings represents an important weakness of this study. However, experience of the single operator who performed all hysteroscopies and the use of a standard report to record abnormal findings limit the impact of such a bias.

The previously published data show large ranges of abnormal finding rates from one study to another (7.2% to 64%) [7–16]. These differences could be explained by the type of hysteroscopic distension medium and/or hysteroscopic technique used, modifying the surgeon's perception of intrauterine filling defects [17]. Results could also be influenced by the characteristics of the population: age of the population, hormonal status, ethnic factor, type of infertility (primary or secondary) and indications for hysteroscopy (infertility alone, association with clinical, echographic or hysterosalpingographic abnormalities, prior to IVF ect.).

Dicker et al. founded higher rates of abnormal findings in elderly women (above 40 years old). Abnormalities such as submucous myomas, endometrial hyperplasia, and polyps were more frequent in this population, while in younger patients other uterine lesions such as adhesions and tubal ostia occlusion were more common [7]. When comparing hysteroscopic abnormalities before and after 38 years of age, Magos et al. [13] did not show a significant difference (51% of abnormal finding before 38 years and 43% after, *P* = .38).This result might be explained by the high rate of endometritis in their population (17.2%), which was more frequently observed in younger woman.

Many studies describe the incidence of abnormal findings with hysteroscopy in infertile women or prior to IVF, but none give the proportion of these women who could benefit from an adapted treatment based on hysteroscopic findings. It is difficult to draw direct connections between hysteroscopic findings and benefits from a specific treatment based on these findings. Treatments for some abnormalities are suspected beneficial in infertile women. These are intrauterine adhesions, congenital uterine malformations, endometrial polyps, and uterine myomas [18]. Chronic endometrial inflammation and micropolyps have also been related to infertility and recurrent miscarriages [19]. 

It is not clear yet if abnormal hysteroscopic findings, by guiding infertility treatments, increase pregnancy rates. In our population we founded abnormal hysteroscopic findings in 40% of the infertile women, and 75% of these abnormalities could be related to infertility and benefit from a specific treatment. La Sala et al. suggest hysteroscopy as a routine exam in infertile woman because it would be economically advantageous, in regard to costs of assisted reproductive technology [14].

## 5. Conclusions

Rates of abnormal findings in unselected infertile patient who underwent diagnostic hysteroscopy ranged from 30% at 30 years to more than 60% after 42 years. Risk of abnormal finding was multiplied by a factor of 1.5 every 5 years. Our data are an additional argument to propose office hysteroscopy as part of first line exams in infertile woman, regardless of age.

## Figures and Tables

**Figure 1:**
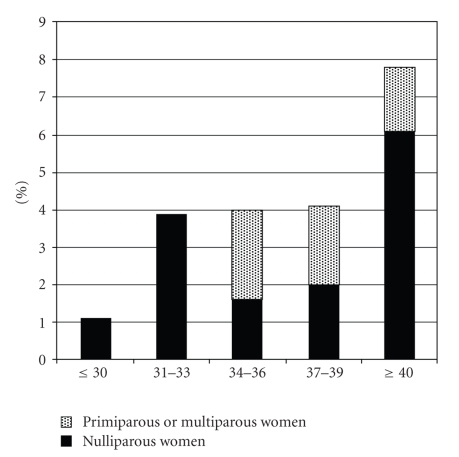
Rates of cervical abnormalities in 557 infertile women during office hysteroscopy according to age.
